# Upper Gastrointestinal Mucosal Injury and Symptoms in Elderly Low-Dose Aspirin Users

**DOI:** 10.1155/2015/252963

**Published:** 2015-01-26

**Authors:** Yuji Shimada, Akihito Nagahara, Mariko Hojo, Daisuke Asaoka, Hitoshi Sasaki, Hiroya Ueyama, Kenshi Matsumoto, Sumio Watanabe

**Affiliations:** Department of Gastroenterology, Juntendo University School of Medicine, 2-1-1 Hongo Bunkyo-ku, Tokyo 113-8421, Japan

## Abstract

*Background*. We investigated the prevalence, symptoms, and QOL impact of esophageal (EI), gastric (GI), and duodenal mucosal injury (DI) individually between low-dose aspirin (LDA) users and nonusers to reveal the clinical features of LDA-related mucosal injury.* Methods*. Data were extracted from the records of subjects who underwent upper gastrointestinal endoscopy at our department between April 2008 and December 2013. Responses from 3162 elderly patients on Frequency Scale for Symptoms of GERD (FSSG) and SF-8 QOL questionnaires (SF-8) were analyzed. FSSG items were classified into total score (TS), reflux score (RS), and dyspepsia score (DS). The SF-8 questionnaire consisted of the physical component summary (PCS) and mental component summary (MCS).* Results*. Prevalence among LDA users and nonusers, respectively, was 9.6% and 10.0% (*P* = 0.83) for EI, 35.9% and 27.5% (*P* = 0.0027) for GI, 3.3% and 3.4% (*P* = 0.84) for DI, and 8.2% and 5.2% (*P* = 0.036) for mucosal injury in 2 or more organs. LDA users diagnosed with EI had significantly lower PCS, LDA users diagnosed with GI had significantly lower DS, and LDA users diagnosed with DI had significantly lower RS and significantly lower MCS.* Conclusion*. These results provide important clinical information indicating that symptom-based management is not appropriate in LDA users regarding upper gastrointestinal mucosal injury.

## 1. Introduction

Low-dose aspirin (LDA) plays an important role in the prevention of atherosclerosis-related diseases through its antiplatelet effects [1, 2]. LDA use is increasing dramatically around the world [1, 3]. In Japan in particular, the number of LDA users is expected to increase due to the country's aging population [4]. However, it is known that in clinical practice LDA causes upper gastrointestinal mucosal damage, which can lead to critical conditions related to ulcer bleeding [5]. Additionally, among elderly patients, mucosal injury can easily develop into serious ulceration and bleeding, asymptomatically [6]. Therefore, it is important to determine precisely where and how frequently mucosal injury occurs in elderly patients taking LDA. For this reason, the current study focused exclusively on elderly patients (>65 years of age).

There have been many reports about LDA-related gastrointestinal complications; however, most of them investigated the relationship between LDA and gastrointestinal bleeding or bleeding risk [7–10]. Other studies have simply focused on the relationship between LDA and upper gastrointestinal mucosal injury, but most did not investigate upper gastrointestinal mucosal injury in individual organs (i.e., esophagus, stomach, and duodenum). We felt that it was important to understand the features of LDA-related mucosal injury, specifically in individual organs, because it would be helpful in identifying the site of mucosal injury in patients taking LDA. Moreover, few studies have investigated subjects diagnosed with mucosal injury in 2 or more organs [11–14].

We also investigated the clinical features of patients with LDA-related upper gastrointestinal mucosal injury based on symptomatology at the time of endoscopy as assessed using questionnaires. It has been documented that continuous LDA treatment leads to worsening of various upper gastrointestinal symptoms [15–17]. Conversely, 1 study reported that there were no significant differences between ulcer and nonulcer patients taking LDA with respect to the frequency and severity of symptoms [11]. Additionally, no studies to date have examined the relationship between taking LDA and quality of life (QOL).

In this study, we investigated the prevalence, severity, and symptoms of esophageal mucosal injury (EI), gastric mucosal injury (GI), and duodenal mucosal injury (DI) in patients taking LDA (LDA users) and not taking LDA (nonusers) to reveal the clinical features of LDA-related upper gastrointestinal mucosal injury, and to reveal the relationship between mucosal injuries among these different organs we evaluated mucosal injury among different organs in each patient.

## 2. Methods

This is a retrospective cross-sectional study performed in a single university hospital between April 2008 and December 2013. The Juntendo University Ethics Committee approved the study protocol. The performance of this study adhered to the principles of the Declaration of Helsinki for medical research involving human subjects.

**Figure 1:**
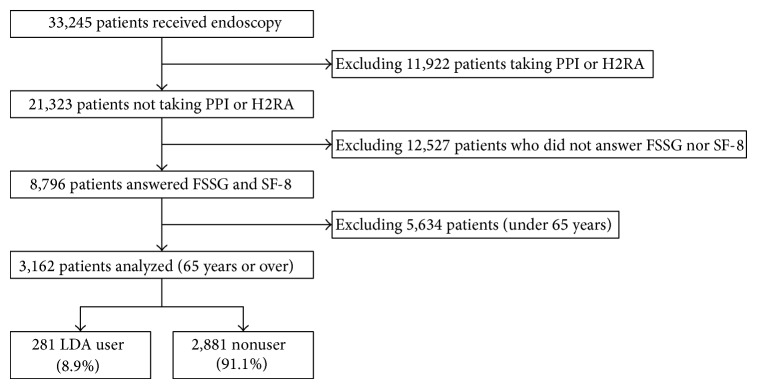
Flow chart of the study patients. PPI = proton pump inhibitor, H2RA = histamine-2 receptor antagonist, FSSG = Frequency Scale for Symptoms of GERD, and SF-8 = Short-Form 8 Health Survey.

Data were extracted from the records of subjects who underwent esophagogastroduodenoscopy (EGD) at our department between April 2008 and December 2013. Of the 33,245 subjects analyzed, 8,796 subjects who had filled in the Frequency Scale for Symptoms of GERD (FSSG) and Short-Form 8 Health Survey (SF-8) questionnaires were selected, after excluding subjects who took a proton pump inhibitor (PPI) or histamine-2 receptor antagonist (H2RA). Among these subjects, we focused on 3,162 subjects who were 65 years of age or over (Figure 1).

We analyzed data for subjects diagnosed endoscopically with esophageal mucosal injury (EI; Los Angeles grade A, B, C, and D esophagitis), gastric mucosal injury (GI; gastric erosions and/or ulcers), or duodenal mucosal injury (DI; duodenal erosions and/or ulcers), individually. Gastric and duodenal mucosal injuries are based on the definition of ulcer classification which was defined by Murakami and Suzuki in 1971 [18].

We compared the prevalence, severity, and symptoms of EI, GI, and DI between LDA users and nonusers to reveal the clinical features of LDA-related upper GI mucosal injury. LDA users were defined as subjects who at the time of EGD had been regularly prescribed aspirin daily (at a dose of 81 mg or 100 mg) because of chronic disease.

To assess the symptoms of patients, we employed the FSSG, which was developed for evaluation of GERD symptoms in Japanese patients and comprises the 12 most frequent symptoms [19, 20]. This questionnaire is useful not only for objectively evaluating the therapeutic response of GERD but also potentially for patients who have GI or DI, because the FSSG items are classified into total score (TS), reflux score (RS) (questions 1, 4, 6, 7, 9, 10, and 12), and dyspepsia score (DS) (questions 2, 3, 5, 8, and 11).

To assess the QOL of patients, the SF-8 questionnaire was used as a comprehensive scale. The SF-8 is an alternative to the SF-36 Health Survey (SF-36) questionnaire and uses 1 question to measure each of 8 SF-36 domains. Since the SF-8 has only 8 questions, it can be completed in 1 to 2 minutes, but it works best both for monitoring population health and for large-scale outcome studies [21]. SF-8 QOL scores were analyzed in terms of the physical component summary (PCS) and mental component summary (MCS).

Statistical analyses were performed by Mann-Whitney *U* test. Two-sided *P* values less than 0.05 were considered statistically significant.

## 3. Results

The study included 281 LDA users (199 men, 82 women; age range 65 to 87 years; mean age 71.7 years) and 2881 nonusers (1617 men, 1264 women; age range 65 to 93 years; mean age 72.7 years).

**Figure 2:**
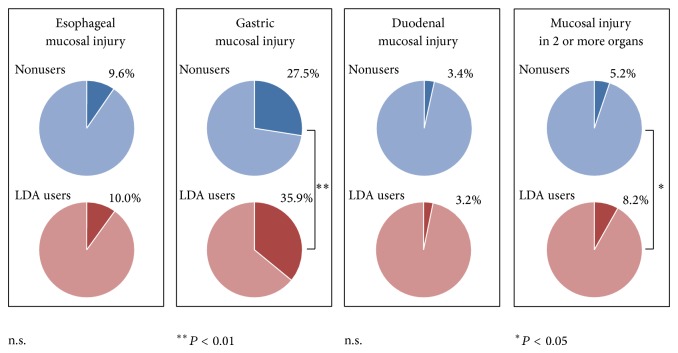
Prevalence of esophageal, gastric, and duodenal mucosal injury and concurrent mucosal injury in 2 or more organs in LDA users and nonusers. Prevalence in LDA users and nonusers was, respectively, 10.0% and 9.6% (*P* = 0.83) for esophageal mucosal injury, 35.9% and 27.5% (*P* = 0.003) for gastric mucosal injury, and 3.2% and 3.4% (*P* = 0.84) for duodenal mucosal injury. The prevalence of mucosal injury in 2 more organs was 8.2% in LDA users and 5.2% (*P* = 0.04) in nonusers. n.s. = not significant.

Figure 2 shows the prevalence of esophageal, gastric, duodenal mucosal injury and concurrent mucosal injury in 2 or more organs in LDA users and nonusers. In LDA users and nonusers, respectively, EI prevalence was 9.6% (*n* = 28) and 10.0% (*n* = 276; *P* = 0.83), GI prevalence was 35.9% (*n* = 101) and 27.5% (*n* = 792; *P* = 0.0027), and DI prevalence was 3.3% (*n* = 9) and 3.4% (*n* = 99; *P* = 0.84). The prevalence of mucosal injury in 2 or more organs concurrently (i.e., EI + GI, EI + DI, GI + DI, or EI + GI + DI) was 8.2% (*n* = 23) in LDA users and 5.2% (*n* = 150; *P* = 0.036) in nonusers.

**Figure 3:**
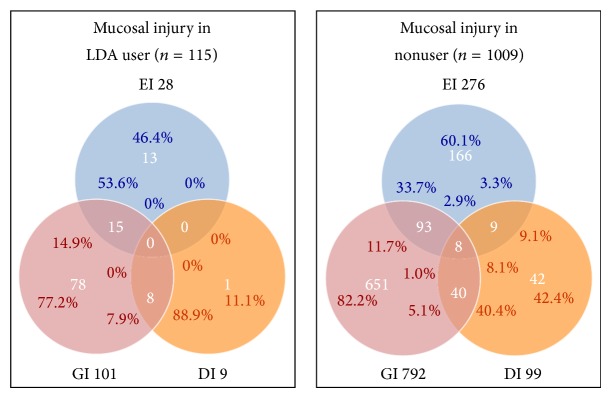
Proportions of patients with different forms of mucosal injury. In patients who had esophageal mucosal injury, the percentage with concurrent gastric mucosal injury was 53.6% for LDA users versus 33.7% for nonusers. In patients who had duodenal mucosal injury, the percentage with concurrent gastric mucosal injury was 88.9% for LDA users versus 40.4% for nonusers. EI = esophageal mucosal injury, GI = gastric mucosal injury, and DI = duodenal mucosal injury.

The proportions of patients with different forms of mucosal injury are shown in Figure 3: In patients who had esophageal mucosal injury, the percentage with concurrent gastric mucosal injury was 53.6% for LDA users versus 33.7% for nonusers. In patients who had duodenal mucosal injury, the percentage with concurrent gastric mucosal injury was 88.9% for LDA users versus 40.4% for nonusers.

Characteristics of subjects with mucosal injury are shown in Table 1. Among subjects with GI, the proportion of men to women was higher for LDA users than nonusers.

**Figure 4:**
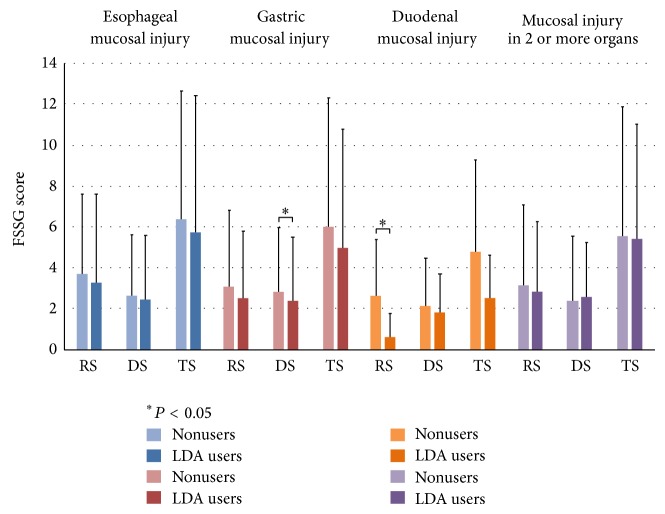
FSSG score of subjects with mucosal injury among LDA users and nonusers. Among LDA users and nonusers, respectively, the scores forTS, RS, and DS were 5.8 ± 6.6 and 6.4 ± 6.3 (*P* = 0.29), 3.3 ± 4.3 and 3.7 ± 3.9 (*P* = 0.17), and 2.5 ± 3.1 and 2.7 ± 2.9 (*P* = 0.46) for EI; 5.0 ± 5.8 and 6.0 ± 6.3 (*P* = 0.05), 2.6 ± 3.2 and 3.1 ± 3.7 (*P* = 0.09), and 2.4 ± 3.1 and 2.9 ± 3.1 (*P* = 0.04) for GI; 2.6 ± 2.1 and 4.8 ± 4.5 (*P* = 0.16), 0.7 ± 1.1 and 2.6 ± 2.8 (*P* = 0.01), and 1.9 ± 1.8 and 2.2 ± 2.3 (*P* = 0.77) for DI; and 5.5 ± 6.3 and 5.6 ± 5.5 (*P* = 0.60), 2.9 ± 3.9 and 3.2 ± 3.4 (*P* = 0.22), and 2.6 ± 3.1 and 2.4 ± 2.6 (*P* = 0.97) for mucosal injury in 2 or more organs. RS = reflux score, DS = dysmotility score, and TS = total score.

**Figure 5:**
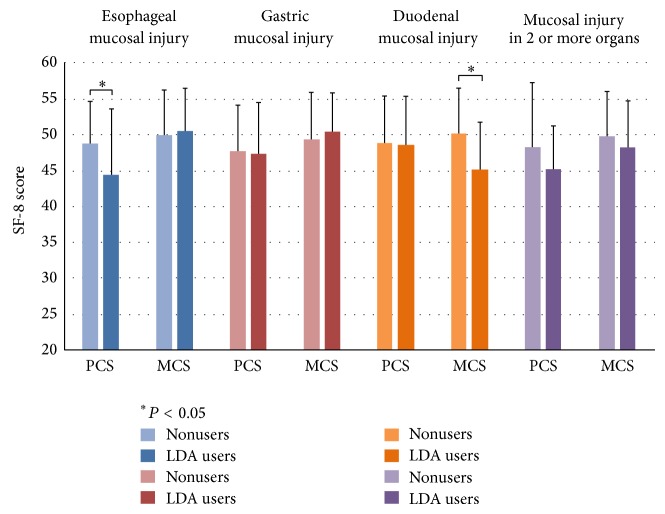
SF-8 score for mucosal injury in LDA users and nonusers. PCS and MCS among LDA users and nonusers were 44.5 ± 9.1 and 48.9 ± 5.8 (*P* = 0.02) and 50.6 ± 5.9 and 50.0 ± 6.3 (*P* = 0.73) for EI; 47.4 ± 7.1 and 47.7 ± 6.4 (*P* = 0.75) and 50.5 ± 5.4 and 49.5 ± 6.6 (*P* = 0.27) for GI; 48.8 ± 6.7 and 49.0 ± 6.6 (*P* = 0.85) and 45.3 ± 6.6 and 50.3 ± 6.3 (*P* = 0.04) for DI; and 45.4 ± 9.1 and 48.3 ± 6.0 (*P* = 0.20) and 48.4 ± 6.3 and 49.8 ± 6.4 (*P* = 0.28) for mucosal injury in 2 or more organs. PCS = physical component score and MCS = mental component score.

Regarding symptoms and QOL, FSSG score of subjects with mucosal injury among LDA users and nonusers is shown in Figure 4, and SF-8 score for mucosal injury in LDA users and nonusers is shown in Figure 5. LDA users diagnosed with GI had significantly lower DS, and LDA users diagnosed with DI had significantly lower RS (Figure 4). LDA users diagnosed with EI had significantly lower PCS, and LDA users diagnosed with DI had significantly lower MCS.

## 4. Discussion

This study shows the features of LDA-related upper gastrointestinal mucosal injury in elderly LDA users and nonusers. According to previous studies of peptic ulcers in patients from Western countries, the prevalence of GI in LDA users has been estimated at 65.2% [11] and 54.3% [22], as compared with 11.8% [12] and 36.7% [23] in Japanese patients. In the present study, the prevalence of GI in LDA users was 35.9% and was statistically significantly higher than in nonusers (27.5%). This result was similar to findings from previous Japanese reports but lower than those from Western reports. Potential reasons for the difference include age range, race, and the rate of* H. pylori *infection [24], but the role of* H. pylori *remains unclear. In our study the proportions of* H. pylori*-positive patients among LDA users and nonusers would likely be similar; however, we did not test to confirm the presence of* H. pylori *infection. Currently, the mechanism of aspirin-induced gastric mucosal damage is thought to be mediated by multiple processes involving either topical or systemic effects [25, 26]; however, their relative contributions to aspirin-related upper gastrointestinal mucosal injury have not yet been fully elucidated. Some reports [27, 28] have suggested that topical effects play a pivotal role in causing GI in the case of aspirin but not in the case of other NSAIDs.

The prevalence of DI in LDA users has been estimated at 24.1% [11] and 21.7% [22] in Western countries, but only 1.3% [12] and 4.7% [23] in Japan. In the present study, the prevalence of DI in LDA users was 3.2% and did not differ significantly relative to nonusers (3.4%). The prevalence in LDA users was similar to that of other Japanese reports and consistent with the trend observed with respect to GI. The mechanisms of aspirin-induced DI differ from those of GI in several respects, because bile acid, enterobacteria, proteolytic enzymes, and toxins can easily penetrate the mucosa and cause DI under conditions in which mucosal permeability is increased [29].

Opinion is divided as to whether or not aspirin is a risk factor for EI. Some multicenter, double-blind, placebo-controlled studies from Western countries have reported that LDA is a risk factor for EI [30, 31]. Conversely, a study from Japan suggested that taking LDA is not a risk factor for EI [14]. In the present study, the prevalence of EI in LDA users was 10.0% and did not differ significantly from that in nonusers (9.6%). The prevalence in LDA users was also similar to previous reports from Japan. However, the reason for the discrepancy in the prevalence of EI in studies from Japan and Western countries remains unclear. It is thought that differences in the study populations, including age range, race, ethnicity, and the rate of* H. pylori *infection, may be responsible [32–35]. The mechanism of aspirin-induced EI remains to be fully elucidated. It is thought that inhibition by aspirin of prostaglandins derived from COX-1 weakens the esophageal mucosal defense system, as is the case with the gastric and duodenal mucosa, and allows agents such as gastric acid, pepsin, and bile salts to enter the esophageal mucosa and cause injury [36].

In the present study, we investigated subjects who were diagnosed with mucosal injury in 2 or 3 organs concurrently. Among LDA users, the prevalence of concurrent EI and GI or concurrent GI and DI was higher than in nonusers. Surprisingly, over half of patients taking LDA who had EI or DI also had concurrent GI. The reason for this result remains unknown. Previous investigators have reported that luminal acidic conditions are a necessary factor for GI and that mucosal injury is induced in an acid-dependent manner [37, 38]. We reasoned that, in such cases, inflow to the duodenum or reflux into the esophagus of excessive acidic gastric contents may subsequently cause mucosal injury.

We also investigated the relationship of LDA with symptoms and QOL. Many studies have examined the relationship between aspirin use and upper abdominal symptoms; however, it remains controversial as to whether or not the administration of aspirin can induce upper gastrointestinal symptoms [39–42]. One important factor to consider is that we focused only on elderly patients in the present study. It is known that advancing age influences visceral sensory functions and changes the degree of various symptoms, but the trend varies by type of symptoms [43–48]. However, in the present study, we found that there was no relationship between LDA and symptoms. In relation to the mechanism of impaired perception of visceral pain in elderly people, Moore and associates [49] pointed out that some age-related differences do in fact exist.

It is known that patients with peptic ulcer have lower QOL than healthy people and that patients taking aspirin experience decreased QOL. However, in our study, no significant differences were found between patients with gastric ulcer or those who did not take aspirin [50, 51]. Among patients with DI, LDA users had significantly lower MCS than nonusers. It has also been reported that GERD patients have lower QOL than do healthy people [52], but there have been no reports about QOL in GERD patients taking aspirin. In the present study, among patients with EI, PCS in LDA users was significantly lower than in nonusers.

We have several limitations. Firstly, we might have a potential bias of patients because this is a retrospective cross-sectional study, retrospective medical record review study, performed in a single university hospital. However, it may represent the common clinical site because we usually perform endoscopy not only for the patients who require detailed examinations or urgent patients, but also for the patients who just want to have a regular checkup. Secondly, only 64.1% of patients answered both FSSG and SF-8 questionnaire. It might introduce bias into the result.

In conclusion, we have demonstrated that the prevalence of gastric mucosal injury in elderly patients taking LDA was significantly higher than in nonusers. However, there was no difference in the prevalence of esophageal or duodenal mucosal injury between elderly patients who took LDA and those who did not. Furthermore, symptoms were not more prevalent in LDA users with upper gastrointestinal mucosal injury versus nonusers. These results provide important clinical information indicating that symptom-based management is not appropriate in elderly LDA users with upper gastrointestinal mucosal injuries. This is the first study to reveal the prevalence, symptoms, and QOL of upper gastrointestinal mucosal injuries in patients taking low-dose aspirin in individual organs at the same time from one same population. It is particularly unique point on this topic. We believe these results will be helpful for clinicians to manage the patients of low-dose aspirin users.

## Figures and Tables

**Table 1:** Characteristics of subjects with mucosal injury in individual organs.

	Esophageal mucosal injury		Gastric mucosal injury		Duodenal mucosal injury		Mucosal injury in 2 or more organs	
	LDA users (*n* = 28)	Nonusers (*n* = 276)	LDA users (*n* = 101)	Nonusers (*n* = 792)	LDA users (*n* = 9)	Nonusers (*n* = 99)	LDA users (*n* = 23)	Nonusers (*n* = 150)
Age (year)	72.2 ± 5.4	71.3 ± 5.3	72.1 ± 5.2	71.3 ± 5.0	71.9 ± 5.2	71.2 ± 5.2	71.4 ± 5.1	71.4 ± 5.2
Gender (M/F) (%)	18/10 (64.3/35.7)	180/96 (65.2/34.8)	79/22 (78.2/21.8)^***^	438/354 (55.3/44.7)^***^	6/3 (66.7/33.3)	76/23 (76.8/23.2)	17/6 (73.9/26.1)	98/52 (65.3/34.7)
BMI (kg/m^2^)	23.1 ± 3.2	23.5 ± 4.0	23.6 ± 3.1^**^	22.6 ± 3.5^**^	24.3 ± 2.4	23.0 ± 3.0	23.3 ± 3.1	24.5 ± 3.3

^**^ *P* < 0.01.

^***^ *P* < 0.001.

Among subjects with gastric mucosal injury, a higher proportion of men to women was evident among LDA users compared with nonusers. Data represent mean ± SD. BMI = body mass index.
